# The use of nitrogen-15 in microbial natural product discovery and biosynthetic characterization

**DOI:** 10.3389/fmicb.2023.1174591

**Published:** 2023-05-10

**Authors:** Kalindi D. Morgan

**Affiliations:** Department of Chemistry and Biochemistry, University of Northern British Columbia, Prince George, BC, Canada

**Keywords:** natural products, nitrogen-15, NMR-guided discovery, mass spectrometry, structure elucidation, biosynthetic characterization, biosynthetic gene clusters

## Abstract

This mini-review covers the use of nitrogen-15 in bacterial and fungal natural product discovery and biosynthetic characterization from 1970 to 2022. Nitrogen is an important element in a number of bioactive and structurally intriguing natural products including alkaloids, non-ribosomal peptides, and hybrid natural products. Nitrogen-15 can be detected at natural abundance utilizing two-dimensional nuclear magnetic resonance and mass spectrometry. Additionally, it is a stable isotope that can be added to growth media for both filamentous fungi and bacteria. With stable isotope feeding, additional two-dimensional nuclear magnetic resonance and mass spectrometry strategies have become available, and there is a growing trend to use nitrogen-15 stable isotope feeding for the biosynthetic characterization of natural products. This mini-review will catalog the use of these strategies, analyze the strengths and weaknesses of the different approaches, and suggest future directions for the use of nitrogen-15 in natural product discovery and biosynthetic characterization.

## 1. Nitrogen-15 in natural products

Nitrogen is often found within structurally interesting and bioactive microbial natural products in varying frequencies and moieties. Natural product classes with significant nitrogen representation include alkaloids, non-ribosomal peptides, polyketide macrolactams, and ribosomally synthesized post-translationally modified peptides (RiPPs). Due to this prevalence, tools and methodologies that can detect nitrogen are of great use to those who study microbial natural products. Important tools are nuclear magnetic resonance (NMR) and mass spectrometry (MS). ^15^N-NMR can be employed strategically for structure elucidation and discovery, while MS methodologies are pivotal for biosynthetic characterization and discovery particularly by detecting nitrogen-15 isotope ratio shifts.

## 2. Nitrogen-15 NMR

### 2.1. Nitrogen-15 NMR background

Nitrogen-15 nuclear magnetic resonance (NMR) spectroscopy is a powerful tool for elucidating natural product structures which contain nitrogen atoms. With steady instrument improvements and new techniques, valuable NMR data can be gathered with increasingly less material (Breton and Reynolds, 2013). Of the commonly found elements in natural products—hydrogens (i.e., protons), carbon, oxygen, nitrogen, sulfur and phosphorous– experiments can be run to directly probe small variations in structural environments for ^1^H, ^13^C,^14^N, ^15^N, and ^31^P nuclei (Parella and Sánchez-Ferrando, 2004; Martin, 2011). Nitrogen has two NMR active isotopes: ^14^N and ^15^N. ^14^N is the more abundant isotope; however, ^14^N is a spin-1 nucleus, and the signal suffers from significant line broadening due to quadrupolar relaxation (Witanowski and Webb, 1972). Alternately, nitrogen-15 is a spin-1/2 nucleus that does not experience significant line broadening, and is thus the more commonly observed nitrogen isotope (Witanowski and Webb, 1972). However, ^15^N has a natural abundance of only 0.36%, making it less sensitive than other more abundant nuclei such as carbon-13 or proton. The ^15^N isotope's negative gyromagnetic ratio (−2.7126) (Martin and Williams, 2010) also results in decreased sensitivity of the ^15^N isotope in NMR experiments in comparison to isotopes with a positive gyromagnetic ratio.

Methods for overcoming both ^15^N and ^13^C signal insensitivities incorporate a suite of two-dimensional (2D) NMR experiments in which the sensitivity of the proton is transferred to the more insensitive nuclei, increasing the signal-to-noise ratio and permitting data analysis of lower-abundance metabolites (Marek and Lycka, 2002; Martin and Williams, 2010). In general, two phenomena are exploited to transfer sensitivity from the proton to insensitive nuclei, such as ^13^C and ^15^N: the nuclear Overhauser effect, and polarization transfer for scalar coupled nuclei (Morris and Freeman, 1979; Freeman, 1997). Sensitivity transfer through the nuclear Overhauser effect is directly related to the gyromagnetic ratio of the observed nuclei. Because of this relationship, the nuclear Overhauser effect is not of value for sensitivity enhancement of the ^15^N nuclei with its negative gyromagnetic ratio (Freeman, 1997). The base experiment for non-selective sensitivity enhancement for scalar coupled nuclei is called the Insensitive Nuclei Enhanced by Polarization Transfer (INEPT) experiment (Morris and Freeman, 1979). Polarization transfer in scalar coupled nuclei is independent of the sign of the gyromagnetic ratio and, as such, is very useful for ^15^N experiments (Morris and Freeman, 1979). One caveat is that because polarization transfer relies on scalar coupling, through-bond connectivity must occur for this enhancement. Usefully, in inverse detected 2D experiments, the ^15^N isotope experiences significant increases in detection sensitivity (Freeman, 1997) due to the large population disparity between ^1^H and ^15^N, which arises from the sizeable gyromagnetic ratio difference between ^1^H (26.7522) and ^15^N (−2.7126) (Marek and Lycka, 2002). With experiments built off the INEPT foundation (Morris and Freeman, 1979), ^15^N is routinely used in NMR methods for elucidating amino acids for protein structure determination (Mulder and Filatov, 2010; Williamson et al., 2014), while usage in small molecule structure determination has varied in the literature (Marek and Lycka, 2002; Martin and Williams, 2010). The low natural abundance can be circumvented by incorporating stable isotope (or labeled) precursors into natural products to increase the ^15^N signal (Bax, 1989; Ohki and Kainosho, 2008; Chokkathukalam et al., 2014; Deev et al., 2017). However, with 2D experiments, natural abundance is usually enough to collect data from pure samples (Martin and Hadden, 2000; Marek and Lycka, 2002). Another barrier to its use has been the historical tendency to report nitrogen referenced to various internal and external references (Witanowski and Webb, 1972; Wishart et al., 1995), which has made it difficult to look to the literature for typical chemical shifts that are widely applicable. Fortunately, over the last several decades, there has been a shift to reference ^15^N NMR spectra to either nitromethane or liquid ammonia set equal to 0 ppm (IUPAC recommendation Harris et al., 2002; Martin and Williams, 2010). Additionally, experimental work has been done to compare the shifts of nitromethane and liquid ammonia, meaning chemical shift values can be easily converted between these two ^15^N references (Witanowski and Webb, 1972; Martin et al., 2007; Martin and Williams, 2010; Martin, 2011). Nitrogen chemical shifts referenced to liquid ammonia tend to have positive signs. Nitromethane is more deshielded than most nitrogens in natural products; this has historically been represented with shielded nitrogens possessing negative values in relation to nitromethane (IUPAC recommendation (Harris et al., 2002). Since about 2015, chemists are reversing that convention in relation to nitromethane (Martin and Williams, 2015). However, because most natural product nitrogens are more shielded than nitromethane, if a negative nitrogen chemical shift is reported, it can be generally assumed the historical conventions are being followed.

### 2.2. Nitrogen-15 NMR in natural product structure elucidation and discovery

**Figure 1:**
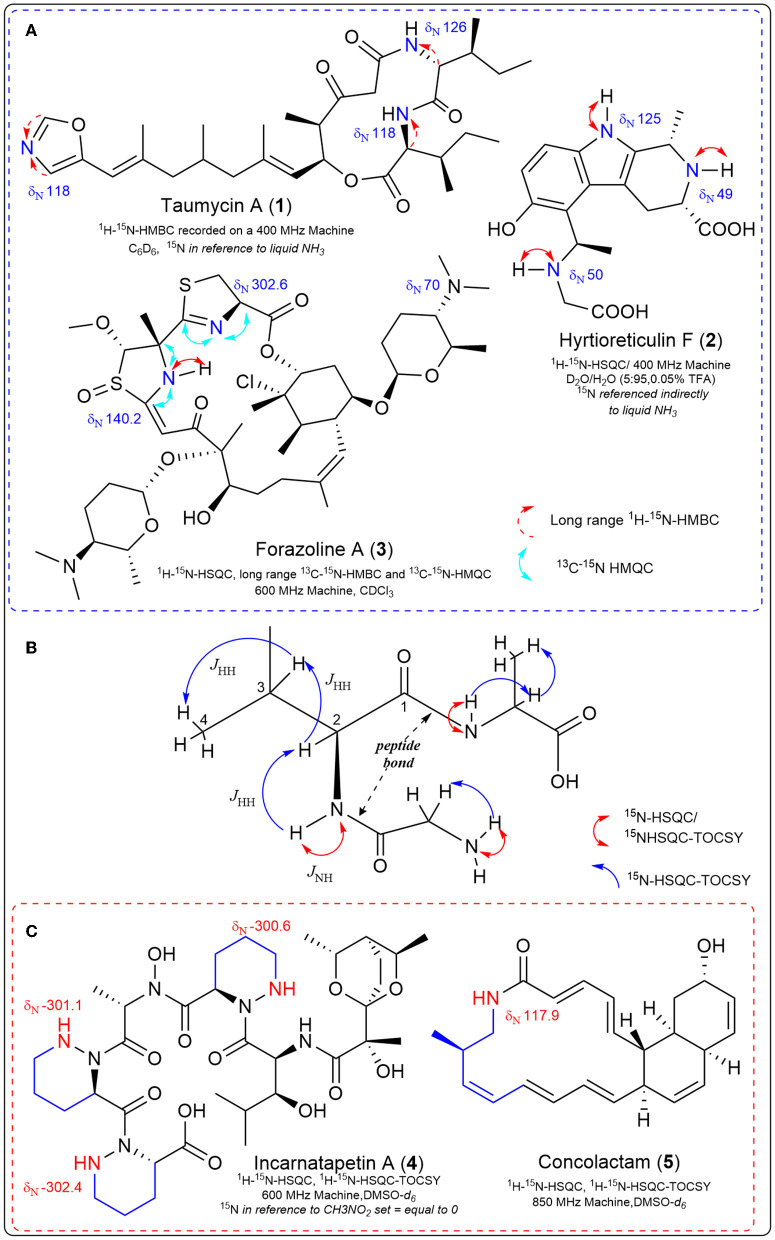
Experimentally determined nitrogen chemical shifts are provided, along with key correlations observed from various nitrogen-15 NMR experiments. **(A)** Select natural products structures that required ^15^N NMR 2D experiments during structure elucidation. **(B)** ^1^H-^15^N HSQC-TOCSY is an NMR experiment that allows us to observe the proton spin system of a single amino acid embedded in a peptide, beginning with the N-H correlation. The carbonyl of the peptide bond provides an insulator functionality that constrains the observation of single amino acid spin systems in the TOCSY spectrum. **(C)** Key examples where ^15^N NMR spectroscopy was integrated with genomic data for natural product discovery.

Two-dimensional (2D) NMR experiments that indirectly detect nitrogen-15 improve the sensitivity limitations due to nitrogen's natural abundance. These experiments can detect one-bond couplings between nitrogen-15 and their attached protons (the sensitive ^1^H-^15^N HSQC/HMQC), detecting two-to-three bond couplings between tertiary nitrogen and protons in the molecule (long-range ^1^H-^15^N HMBC) (Martin and Hadden, 2000; Martin et al., 2007; Martin and Williams, 2010); and even detect carbon-13-nitrogen-15 correlations (^13^C-^15^N HSQC) with appropriate labeling. These 2D ^15^N NMR experiments are invaluable in solving complex bioactive natural product structures (Figure 1). Examples include the taumycins A (**1**) and B (Bishara et al., 2008), hyrtioreticulin F (**2**) (Imada et al., 2013) and coibamide A (Medina et al., 2008). The structure of the polyketide, forazoline A (**3**), was solved (after stable isotope feeding) utilizing the first example of carbon-13-nitrogen-15 direct detection via ^13^C-^15^N HSQC (Wyche et al., 2014). This same experiment proved vital in the recent structure elucidation of the structurally-unprecedented ecteinamine after discovery using an integrated genomic-metabolomic strategy by the Bugni group (Wu et al., 2022).

These 2D nitrogen-15 experiments can also be used in the discovery process, as first exemplified in 2007 in the genomisotopic approach developed by the Gerwick lab. The genomisotopic approach used ^15^N isotopically labeled precursors as predicted by *in silico* adenylation domain specificity of a genetically encoded non-ribosomal peptide to link orphan secondary metabolite gene clusters to the putatively produced natural product (Gross et al., 2007). In this method, use of the ^1^H-^15^N HMBC experiment to identify a ^15^N labeled isoleucine in tandem with bioassay-guided fractionation led to the successful isolation and structure elucidation of orfamide A representative of a new peptide class.

In fact, one advantage of ^15^N NMR is the large spectral range (~900 ppm), such that different nitrogen-containing functional groups can have widely dispersed ^15^N chemical shifts (Williamson et al., 1999). Due to fewer nitrogen atoms (compared to protons or carbons) in small molecule natural products, ^15^N spectra tend to be less crowded. Specific experiments designed to use this larger spectral dispersion to provide a means of separating crowded proton and carbon signals include the ^1^H-^15^N-HSQC-TOCSY and ^1^H-^15^N-HSQC-NOESY (Williamson et al., 1999). The ^1^H-^15^N-HSQC-TOCSY experiment allows observations of proton spin systems which are ultimately connected to a ^15^N resonance, that is, the set of scalar-coupled protons adjacent to a nitrogen-attached proton (Figure 1). ^1^H-^15^N HSQC experiments can be used to link a primary or secondary nitrogen with its attached proton, which, due to the large dispersion of nitrogen signals, allows observation of spin systems outside the crowded proton spectral window (Williamson et al., 1999). The TOCSY portion of the ^1^H-^15^N HSQC-TOCSY allows observation of magnetization transfer through scalar-coupled protons to observe a complete amino acid ^1^H spin system in peptide natural products (Figure 1). Recently these spectroscopic experiments have been integrated with genomic information to discover piperazic acid (Piz)-containing polyketide-nonribosomal peptides (PK-NRP) (Morgan et al., 2020, 2022a,b). and macrolactams (Shin et al., 2023) (Figure 1). In both cases, the dispersion of the nitrogen signals allowed detection of the predicted genetically encoded chemical structures in crude microbial extracts, significantly speeding up the discovery process. ^15^N NMR was the ideal tool to uncover genomically encoded Piz-containing natural products because Piz contains a unique nitrogen chemical shift and N-H correlation (Williams et al., 2011) distinct from other amides within peptides. Thus, we utilized the sensitive ^15^N-HSQC experiment to quickly screen microbial extracts obtained from small amounts of bacterial cultures fed with the stable isotope Piz-precursor ^15^N_2_ L-Orn (Morgan, 2021). After quickly screening through dozens of ^15^N_2_ L-Orn labeled microbial extracts, the less sensitive ^1^H-^15^N-HSQC-TOCSY was applied for unequivocal detection of piperazic acid-containing extracts that aligned with genetically encoded chemical structures of interest (Morgan et al., 2020). We then conducted microbial scale-up, with efficient isolation guided by the ^1^H-^15^N-HSQC-TOCSY Piz signal leading to the cytotoxic incarnatapeptin B, the related incarnatapeptin A (**4**) and the piperazic-acid containing dentigerumycins G and H (Morgan et al., 2020, 2022a,b). For the discovery of the novel and cytotoxic muanlactam and antibacterial concolactam (**5**) by the Oh group, the presence and expression of genetically encoded macrolactams was detected through PCR analysis of an in-house gDNA library utilizing macrolactam-specific primers (Shin et al., 2023). Then, bacterial strains identified in the PCR experiments were fed the stable isotope ^15^NH_4_Cl to enrich all amide bonds in crude extracts and permit detection of the macrolactams utilizing ^1^H-^15^N-HSQC-TOCSY NMR experiments. After this detection, scale-up provided efficient discovery of the muanlactam, **5** and structural reassignment of salinilactam (Shin et al., 2023). The application of ^1^H-^15^N-HSQC-TOCSY to the discovery of polyketide macrolactams by the Oh group provides strong support for the power of this method as the novel macrolactams contain but one nitrogen each.

## 3. Nitrogen mass spectrometry

### 3.1. Nitrogen mass spectrometry background

As discussed above, nitrogen has two stable isotopes, nitrogen-14 and nitrogen-15 (^15^N), with 99.635% and 0.365% natural abundance, respectively (Wieser and Brand, 2017). Due to the low natural abundance of ^15^N, precursors enriched in ^15^N have been used in biosynthetic characterization to study the metabolic pathways of living organisms for a century (Rinkel and Dickschat, 2015). The most common application of ^15^N is in metabolic labeling experiments, in which the organism is grown in a medium containing ^15^N-labeled compounds, such as ammonium or nitrate. These labeled compounds are then incorporated into the organism's biomass and metabolic intermediates, and the distribution of ^15^N can be used to trace the metabolic pathways of the organism. Originally, isotope ratio mass spectrometry (IRMS) and degradation prior to mass spectrometric analysis were used to assess distribution of incorporated nitrogen-15 in metabolic pathways (Kahn et al., 2002). As mass spectrometers technology developed with increased sensitivity, techniques like ESI-MS began to be utilized for detecting isotopic ratios patterns between ^14^N and ^15^N to detect ^15^N enrichment in metabolic labeling (Kahn et al., 2002; Wieser and Brand, 2017). The advantage of utilizing mass spectrometry (MS) combined with stable isotopes for biosynthetic characterization is the higher sensitivity of the MS instrumentation (Chokkathukalam et al., 2014).

### 3.2. Nitrogen-15 MS for biosynthetic characterization and discovery

**Figure 2:**
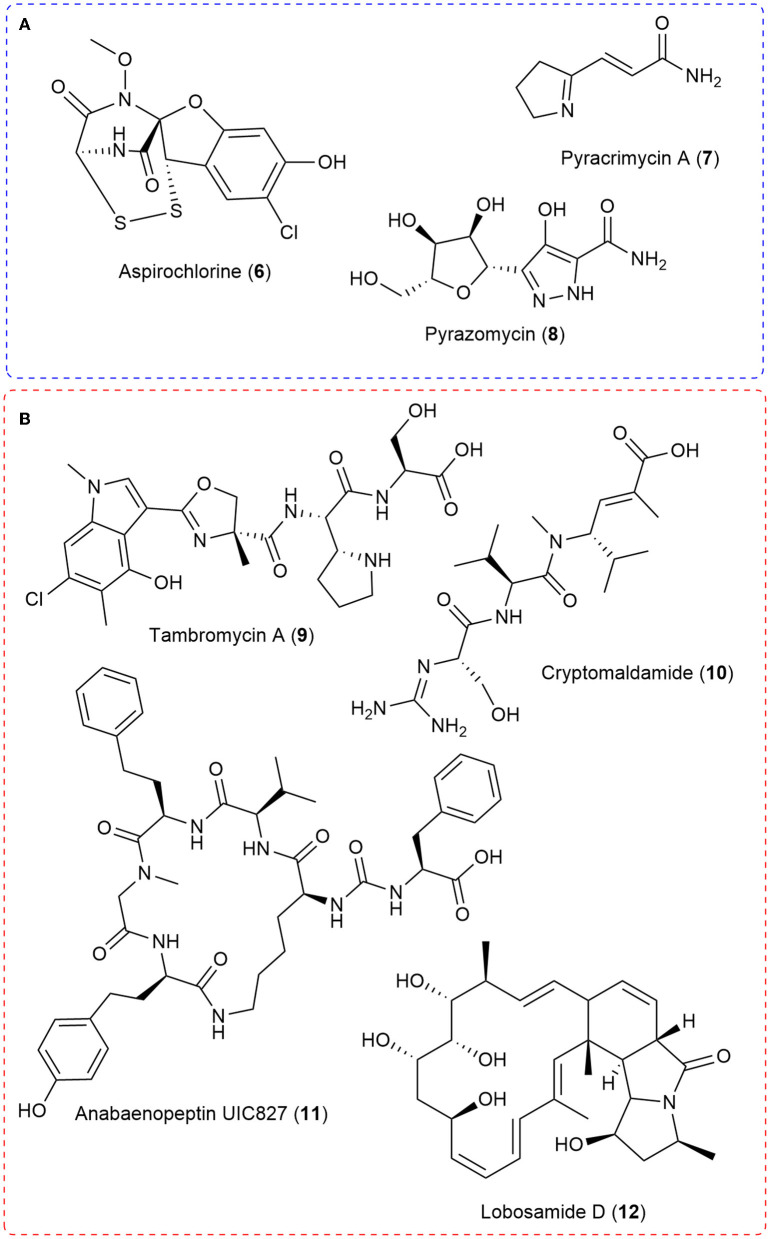
**(A)** Select natural products examples where stable isotope feeding of ^15^N-labeled precursors allowed the use of MS isotopic ratios to characterize all or key components of the biosynthetic pathway. **(B)** A selection of natural products discovered through integrated genomics and MS metabolomic strategies in which ^15^N played a crucial role.

Feeding specific ^15^N-enriched precursors has been used in conjunction with mass spectrometry for natural product biosynthetic characterization (Feng et al., 2009; Rinkel and Dickschat, 2015). By feeding these specific stable isotope precursors enriched with ^15^N into a natural product and then analyzing the resulting isotopomers and isotopic ratios with mass spectrometry, it is possible to determine the biosynthetic pathway of the natural product (Figure 2). Enrichment with ^15^N typically involves the incorporation of a precursor labeled with the stable isotope. In mass spectrometry, the incorporation of a ^15^N-enriched precursor into a molecule can be detected through the observation of isotopic peaks in the mass spectrum. When a molecule enriched with ^15^N isotopes is ionized in the mass spectrometer, it produces a series of isotopic peaks with increased m/z values compared to the peaks of the unlabeled molecule. The number of ^15^N atoms in the molecule determines the spacing between the isotopic peaks. For example, if a molecule with three nitrogen atoms incorporates ^15^N into all three positions, the isotopic peaks will show an increase of three mass units higher than the corresponding peaks of the unlabeled molecule. The increased spacing between the isotopic peaks provides evidence that the molecule has incorporated ^15^N. Additionally, the relative abundance of the isotopic peaks can provide information about the extent of incorporation of the ^15^N-labeled precursor. The higher the relative abundance of the isotopic peaks, the greater the extent of incorporation of the ^15^N-labeled precursor into the molecule.

For example, the biosynthesis of aspirochlorine (**6**) was solved by feeding the stable isotope enriched ^13^C_2_-^15^N-Gly to the producing *Aspergillus oryzae* strain and demonstrating via liquid chromatography-mass spectrometry that only the carbon-13 was incorporated into aspirocholine. This result helped demonstrate that phenylalanine was being transformed into a glycine-like moiety and that glycine was only utilized during N-methylation (Chankhamjon et al., 2014). The Charusanti group recently established the biosynthesis of the antibiotic pyracrimycin A (**7**) in a *Streptomyces* strain utilizing the isotopic ratios in MS analysis after feeding with ^15^N-proline and ^15^N-^13^C_3_-serine, together and individually (Nielsen et al., 2022). ^15^N-enriched precursors were utilized to demonstrate that αNH_2_ nitrogen atom of glutamic acid was involved in forming the nitrogen-nitrogen bond into the rare C-nucleoside antibiotic pyrazomycin (**8**) and point to a new nitrogen-nitrogen bond forming enzyme PyrN (Zhao et al., 2020). More recently, detailed bioinformatic analysis in tandem with feeding synthesized ^15^N-labeled Piz to the **4** producing *Streptomyces incarnatus* NRRL8089 strain followed by high resolution mass spectrometry isotopic ratio analysis demonstrated that free piperazic acid residues are most commonly incorporated into growing non-ribosomal peptide chains (Wei et al., 2022).

Another strategy that has been recently developed is utilizing ^15^N-labeled stable isotope precursors in integrated genomic and mass spectrometry metabolomic approaches (Chokkathukalam et al., 2014). This integration of ^15^N with genomic and metabolomic approaches has allowed the discovery of novel compounds and importantly, the linking of natural product structures with their producing biosynthetic gene clusters (Figure 2). When coupled with metabolomic data analysis methods such as multivariate statistical analyses or molecular networking, strains or extracts with potentially novel components can be prioritized for investigation (Hou et al., 2012; Covington et al., 2017). This type of approach has seen the discovery of molecules such as tambromycin (**9**) (Goering et al., 2016) and columbamides (Kleigrewe et al., 2015) and often linked them to their gene cluster using ^15^N-labeled precursors and MS analysis. The structure of **9** was discovered using the metabologenomics approach whereby statistical analysis of the data from simultaneous bioinformatic analysis of 172 draft genomes and MS analysis of extracts from the corresponding bacteria in four media led to an *m/z* value for an unknown molecule that correlated with several bacteria carrying the same gene cluster. Scale-up allowed isolation and structure elucidation of the novel cytotoxic non-ribosomal peptide, tambromycin (**9**). Stable isotope feeding of ^15^N_1_-Lysine (as compared to unlabeled media) was followed by MS isotopic analysis to link tambromycin with its' gene cluster. Next, the Gerwick Lab discovered both columbamides and cryptomaldamide (**10**) integrating genomic and MS analysis. The columbamides, a mild cannabinomimetic, were also discovered by matching molecular networks of MS data from cyanobacterial extracts with putative gene clusters obtained from their draft genomes (Kleigrewe et al., 2015). In the case of **10**, they used MALDI-MS after ^15^N-Nitrate feeding and noticed a 5 dalton shift in the *m/z* value of interest after 4 days of growth. In both cases, they used MS to guide isolation and 1D and 2D NMR to solve the final structures (Kinnel et al., 2017). Building from these ideas, the Orjala lab utilized shifts in MS isotopic ratio after comparing with data from unlabeled media extracts with 99% ^15^N-nitrate labeled media extracts to match the *m/z* value with isotopic shifts indicative of ^15^N incorporation with predicted nitrogen rich gene clusters from sequenced cyanobacteria. Through this approach, they isolated four peptides, two known and two unknown. They named the unknown peptides anabaenopeptin UIC827 (**11**) and nostopyrrolidonamide after solving their structures with NMR and MS/MS data (May et al., 2020). Finally, the Medema and Linington labs have recently developed IsoAnalyst which can analyze MS data of extracts arising from isotope labeling, including ^15^N-labeled precursors, and connect them with their predicted biosynthetic gene clusters. The developers used IsoAnalyst to match multiple gene clusters with their chemical phenotype and discovered lobosamide D (**12**) after structure elucidation with 1D and 2D NMR (McCaughey et al., 2022). In conclusion, using ^15^N-labeled stable isotope precursors in integrated genomic and MS metabolomic approaches has proven to be a successful strategy for discovering novel compounds and linking natural product structures with their producing biosynthetic gene clusters.

## 4. Discussion

Due to their utility, both ^15^N-NMR and MS have been incorporated into various methodologies and strategies to solve natural product structures, to uncover novel natural product structures, to reveal the biogenesis of the natural products, and to link biosynthetic gene clusters to specific natural products. Indeed, nitrogen-15 NMR is a premier tool due to nitrogen's presence in many of the most structurally interesting and bioactive natural products. Nitrogen-15 NMR experiments are inconsistently used for structure elucidation purposes, perhaps because of the historical challenges and the low natural abundance. However, experiments by Gary Martin of Merck (amongst others) demonstrate that inversely detected nitrogen-15 2D data can be collected at natural abundance for even around 1 mg of pure sample (Martin and Hadden, 2000). ^15^N NMR as a discovery tool has produced structurally interesting and bioactive products from its first use by Gross et al. (2007), to the discovery of the incarnatapeptins (Morgan et al., 2020), to the most recent use by Oh et al. combining genomics and spectroscopic tools (Shin et al., 2023). The application of the ^15^N-HSQC-TOCSY experiments for targeted and efficient discovery approaches for both polyketide-nonribosomal peptide hybrids and macrolactams demonstrates that these approaches are worth considering for a wide range of nitrogen-containing natural products. Moreover, the structural revision of salinilactam during the discovery process by Oh et al., demonstrates that these 2D nitrogen-15 NMR experiments are a critical tool for structure elucidation of natural products. NMR instrumentation now routinely come equipped with the ability to detect nitrogen-15. With these modern enhancements, obtaining 15N NMR 2D experiments during dataset collection for all small molecule natural products should be habitual. Furthermore, if novel structures report nitrogen, journals and editors could require 2D nitrogen-15 NMR experiments alongside the proton and carbon spectra whenever not prohibited by older equipment.

Stable isotope ^15^N labeling combined with MS is a powerful tool for biosynthetic characterization and integrated MS-metabolomic and genomic approaches. These strategies have been useful in uncovering new chemistry with a multitude of MS instrumentation. Perhaps more importantly, ^15^N MS is an invaluable tool in biosynthetic characterization, from how molecules like aspirocholorine are made to how the intriguing non-ribosomal piperazic acid residue is incorporated in the building of natural products. Tools such as IsoAnalyst will surely become indispensable for linking genotype to phenotype. However, there are still some significant limitations when using mass spectrometry as a discovery tool, such as varying tendencies of molecules to ionize, media interference in crude samples (Watrous et al., 2012), unpredictable fragmentation patterns of some natural product classes (Liu et al., 2009; Novák et al., 2015; Townsend et al., 2018), and the need to use NMR in order to determine the final structure usually. At the same time, NMR usually suffers from sensitivity limitations. Therefore, just as MS and NMR data are utilized together to solve natural product structure *de novo*, so could the discovery process be amplified utilizing approaches that integrate the ability of ^15^N NMR to detect precise chemical environments with the sensitivity of MS data and the clarity of isotopic ratio shifts. As stable isotopes are increasingly included for structure elucidation, discovery, and gene cluster linking, the community could consider other tools to exploit the presence of precisely included ^15^N and ^13^C isotopes, such as increased usage of 2D ^15^N NMR experiments and analysis of carbon-nitrogen NMR splitting patterns.

## Author contributions

The author confirms being the sole contributor of this work and has approved it for publication.
